# Extracellular AMP Suppresses Endotoxemia-Induced Inflammation by Alleviating Neutrophil Activation

**DOI:** 10.3389/fimmu.2020.01220

**Published:** 2020-07-07

**Authors:** Ye Hua, Dadong Liu, Danyi Zhang, Xu Wang, Qing Wei, Weiting Qin

**Affiliations:** ^1^Institute of Oncology, Affiliated Hospital of Jiangsu University, Zhenjiang, China; ^2^Department of ICU, Affiliated Hospital of Jiangsu University, Zhenjiang, China; ^3^Department of Laboratory Medicine, Affiliated People's Hospital Jiangsu University Zhenjiang, Zhenjiang, China; ^4^Department of General Surgery, Affiliated Hospital of Jiangsu University, Zhenjiang, China; ^5^State Key Laboratory for Oncogenes and Related Genes, Shanghai Cancer Institute Shanghai Jiao Tong University, Shanghai, China

**Keywords:** AMP, A1R, p38 MAPK, neutrophils, endotoxemia

## Abstract

Intracellular adenosine monophosphate (AMP) is indispensable for cellular metabolic processes, and it is interconverted to ADP and/or ATP or activates AMP-activated protein kinase (AMPK). However, the specific biological function of extracellular AMP has not been identified. We evaluated the effect of extracellular AMP using *in vivo* and *in vitro* models of endotoxemia. We found that AMP inhibited inflammation and neutrophil activation in lipopolysaccharide (LPS)-induced endotoxemic mice. The effects of extracellular AMP were abolished by an adenosine 1 receptor (A1R) antagonist but were not influenced by inhibiting the conversion of AMP to adenosine (ADO), indicating that AMP inhibited inflammation by directly activating A1R. In addition, *in vitro* experiments using LPS-stimulated mouse neutrophils showed that AMP inhibited LPS-induced reactive oxygen species (ROS) production, degranulation, and cytokine production, while the effects were reversed by an A1R antagonist. Further research showed that AMP regulated LPS-stimulated neutrophil functions by inhibiting the p38 MAPK pathway. These findings were also confirmed in primary neutrophils derived from healthy human blood. Moreover, we collected serum samples from septic patients. We found that AMP levels were increased compared with those of healthy volunteers and that AMP levels were negatively correlated with disease severity. Together, these data provide evidence that extracellular AMP acts on A1R to suppress endotoxemia-induced inflammation by inhibiting neutrophil overactivation and that the p38 MAPK signaling pathway is involved.

## Introduction

Sepsis-induced acute and excessive inflammatory responses in patients still cause high mortality despite advanced supportive care and clinical therapeutic intervention (1). Immune cells are thought to play a critical role in responses to bacterial infections during sepsis. Neutrophils are the first mediator to enter an affected area during sepsis. Although neutrophils have beneficial effects in eradicating microbial infections, excessive neutrophil activation, with the resultant release of proinflammatory mediators, results in tissue injury and contributes to the development of organ dysfunctions (2, 3). Targeting neutrophil activation is a potential therapeutic strategy to reduce host tissue damage and organ failure in sepsis patients (4).

Intracellular adenosine monophosphate (AMP) is a key cellular metabolite that regulates energy homeostasis and signal transduction (5). By activating AMP-activated protein kinases (AMPKs), intercellular AMP plays critical roles in reprogramming metabolism and regulating growth and has recently been connected to cellular processes, including autophagy and cell polarity (6–8). During cell activation or death, intracellular AMP is directly released into the extracellular environment or hydrolyzed from ATP by ATP hydrolytic enzymes (9). Through the action of ecto-5′-nucleotidase (NT5E, CD73), extracellular AMP is then converted to adenosine (ADO) (10). Extracellular ATP and ADO are well-recognized purinergic signaling molecules that activate P2Rs and P1Rs, respectively. Notably, P2R- and P1R-mediated purinergic signaling frequently shows opposing effects in terms of modulation of immune cell functions: ATP-mediated P2 receptor signaling prevalently facilitates immune cell activation, whereas ADO-mediated P1R signaling mostly restricts immune cell activation (11). However, until now, the role of extracellular AMP in immune responses and endotoxemia has been unclear. Thus, in the present study, we investigated the effects of extracellular AMP on an LPS-induced mouse endotoxemia model. Then, the effects and mechanisms of AMP on LPS-stimulated mouse and human neutrophils were studied. Finally, the levels of AMP in septic patients were also evaluated.

## Materials and Methods

### Ethics Approval and Consent to Participate

This study was approved by the Medical Ethical Committee of Jiangsu University. Blood specimens were obtained from the cubital veins of septic patients and healthy drug-free donors after receiving written informed consent. Consent for the use of these samples was given by the Medical Ethical Committee of Jiangsu University. All of the experiments were performed in accordance with the approved guidelines.

### Clinical Settings

The clinical study was a prospective case–control study. Subjects with sepsis were included. All septic patients were recruited from the intensive care unit (ICU) of the Affiliated Hospital of Jiangsu University. Healthy volunteers were randomly recruited from healthy patients who underwent routine physical examinations. The inclusion criteria for patient selection were (i) clinical evidence of infection, (ii) hyperthermia (elevated body temperature (>38) or hypothermia [lower body temperature (<35)], (iii) tachycardia [increased heart rate (>100 beats/min)], (iv) tachypnea [rapid breathing (>30 breaths/min)], and (v) evidence of inadequate organ function of perfusion within 12 h of enrollment. The exclusion criteria were (i) patients older than 80 years, (ii) cardiac failure (class III or IV), (iii) liver insufficiency, (iv) immunosuppression (a positive HIV and HBs Ag viral serological result and cancer), and (v) prolonged antibiotic therapy. Septic patients who were admitted to the ICU and fulfilled the inclusion and exclusion criteria, as well as the surviving sepsis guidelines from October 2016 to June 2019, were selected (12).

Data collection included general parameters such as age, gender, and severity score, including the Acute Physiology and Chronic Health Evaluation II, sequential Organ Failure Assessment, absolute lymphocyte count, absolute neutrophil count, length of stay in the ICU, 28-day mortality, admission ICU diagnosis, and comorbidities.

Healthy volunteers, in which the absence of evidence of diseases was evaluated by clinical history and laboratory studies, served as controls.

### Blood Samples

Blood samples were collected from septic patients and healthy volunteers. Whole blood samples were collected, and serum samples were obtained after centrifugation at 4°C and 3,000 rpm for 10 min. Then, the serum samples were aliquoted and stored at −80°C for further analysis.

### Materials

LPS (*Escherichia coli* O55:B5), AMP, and fetal bovine serum (FBS) were purchased from Sigma-Aldrich. HBSS (1 × or 10 ×) with or without Ca^2+^ and Mg^2+^, RPMI 1640 medium, and agarose were purchased from Life Technologies. Antibodies against p38 mitogen-activated protein kinase (MAPK) (polyclonal, 9212) and p–p38 MAPK (clone: 12F8) and the p38 MAPK inhibitor SB203580 were purchased from Cell Signaling Technology. Ecto-5′-nucleotidase inhibitor was purchased from Sigma (St. Louis, MO). The A1R agonist [(±)-5′-chloro-5′-deoxy-ENBA], A1R antagonist (SLV 320), CD39 inhibitor sodium metatungstate (POM-1), ADA inhibitor erythro-9-(2-hydroxy-3-nonyl) adenine (EHNA), and non-selective P2X inhibitor pyridoxal phosphate-6-azo (benzene-2,4-disulfonic acid) tetrasodium salt hydrate (PPADS) were purchased from Tocris Bioscience (Ellisville, MO, USA). Non-selective P2Y inhibitor RB2 was purchased from ICN Biochemicals (Aurora, OH, USA). All other chemicals were of reagent grade and obtained from Sigma unless otherwise stated.

### Animal Models and Treatments

C57BL/6 male mice were used to establish LPS-induced endotoxemia (10 mg/kg, i.p.). In the endotoxemia model, C57BL/6 mice were pretreated with the A1R antagonist (1 mg/kg, i.p.) and ecto-5′-nucleotidase inhibitor (50 μg/kg, i.p.) 10 min before LPS (10 mg/kg) and AMP (50 mg/kg, i.p.) injections. Moreover, the control group received the equivalent of vehicle (PBS, used for dissolution of LPS). Eight hours later, the mice were euthanized, and the serum and lung tissues were collected for subsequent experiments.

### Assessment of Neutrophil Infiltration in the Lung

Lung tissue samples were fixed in 10% formalin without inflating for at least 24 h. Then, the EP tube containing the lung tissue for 4 min was centrifuged, the supernatant with a pipette was suck up, the preheated agarose solution was added, and the agarose was waited for solidification. Finally, the agarose solid was removed from the EP tube and put in the dehydration box. The dehydration box was put in the dehydration machine in order to carry out dehydration with gradient alcohol, in detail 75% alcohol 4 h, 85% alcohol 2 h, 90% alcohol 2 h, 95% alcohol 1 h, anhydrous ethanol I 30 min, anhydrous ethanol II 30 min, alcohol benzene 5–10 min, xylene I 5–10 min, and xylene II 5–10 min. Then, the dehydrated samples were put into the melted paraffin and the wax-soaked tissue embedded using the embedding machine. The lung tissue embedded in paraffin was sectioned at 5 μm for routine histology. Then, the paraffin-embedded lung tissue sections were subjected to H&E staining. Histology of the lung was analyzed for the following: alveolar and capillary edema, intravascular and peri-bronchial influx of inflammatory cells, thickness of the alveolar wall, and hemorrhage. The items were semiquantitatively scored as none, minimal, light, moderate, or severe (score 0, 1, 2, 3, or 4, respectively) by a pathologist blinded to the experimental group. The lung injury score was obtained by averaging the scores from the animals within each group.

Myeloperoxidase (MPO) activity in the lung tissues was detected according to the manufacturer's instructions (Nanjing Jiancheng Bioengineering Institute, Jiangsu, China). In brief, equal weights of lung tissue from each group were homogenized in ice-cold 0.9% NaCl to yield a 10% (w/v) homogenate. The homogenates were then cleared by centrifuging at 9,000 × g at 4°C. Aliquots (0.3 ml) were added to a 2.3-ml reaction mixture containing 50 mM potassium phosphate buffer, o-dianisidine, and 20 mM H_2_O_2_ solution. One unit of enzyme activity (expressed as U/g tissue) was defined as the amount of MPO required to cause a change in absorbance measured at 460 nm for 3 min.

Neutrophil infiltration was also detected by using flow cytometry. Briefly, lungs of mice from different treatments were harvested without flushing and then minced and incubated at 37°C in an enzyme cocktail of RPMI containing 2.4 mg/ml collagenase I and 20 μg/ml DNase (Invitrogen), and then mashed through a 70-μm nylon cell strainer (BD Falcon). Single-cell suspensions of the lung tissue were washed twice with ice-cold PBS and resuspended in PBS containing 5% FBS. The appropriately conjugated fluorescent antibodies (anti-LY6G-APC-Cy7, clone: 1AB) were added and incubated on ice for 30 min in the dark. The cells were washed twice with ice-cold PBS, and the resuspended cell pellets were measured by flow cytometry.

### ELISA

ELISA was used to measure cytokines in serum from septic patients and mice in each group and in the culture supernatant of mouse neutrophils after the indicated treatments. Interleukin 1 (IL-1β), tumor necrosis factor (TNF-α), interleukin 6 (IL-6), and human interleukin 8 (IL-8) were detected according to the manufacturer's instructions (Qiaoyi, Shanghai, China). The kit contained a specific antibody immobilized on a 96-well microtiter plate that bound IL-1β/TNF-α/IL-6/IL-8 in the aliquot and a second enzyme-conjugated specific antibody. Following several washings to remove unbound substances and antibodies, a substrate solution was added to the wells. Color development was stopped by sulfuric acid, and optical density was determined at 540 nm with the correction wavelength set at 570 nm in an ELISA plate reader. Results were calculated on a standard curve concentration and multiplied for the dilution factor. Cytokine levels were expressed as pg/mL.

### Isolation and Preparation of Mouse Bone Marrow Neutrophils and Human Neutrophils

Mouse bone marrow neutrophils were isolated as previously described (13). The mice were euthanized, and the bone marrow was harvested from femurs and tibias. The bone marrow was collected in 50-ml centrifuge tubes through a 70-μm cell strainer via perfusion of each bone with a neutrophil isolation buffer. Marrow cells were pelleted in a centrifuge and resuspended in 2 ml of neutrophil isolation buffer (HBSS containing 0.1% bovine serum albumin). Then, the cell solution was placed onto a discontinuous Percoll gradient consisting of a stock Percoll solution diluted to 78, 69, and 52% in HBSS and centrifuged at 1,500 g at 4°C for 30 min. Neutrophils localized to a band between the 78 and 69% layers. This band was collected and washed with a neutrophil isolation buffer and suspended in RPMI 1,640 containing 1% fetal bovine serum. The purity was > 97% as detected by flow cytometry and an APC-Cy7-labeled anti-LY6G antibody.

Human peripheral blood was collected from healthy individuals and septic patients, and neutrophils were isolated using Ficoll/Hypaque centrifugation, as previously described (14). Briefly, blood was mixed with an equal volume of dextran (3% in HBSS) and incubated for 30 min at room temperature. The supernatant was collected and layered on top of Ficoll, followed by centrifugation. The neutrophil-containing pellet was resuspended in 3 ml water (sterile ddH_2_O) for 30 s to facilitate erythrocyte lysis. Isotonicity was restored by the addition of 3 ml 2 × HBSS. The neutrophil pellet was then washed three times with Hank's balanced salt solution and resuspended in RPMI 1,640 containing 1% fetal bovine serum. The purity was > 97% as detected by flow cytometry and the FITC-labeled anti-CD66b (clone: G10F5) antibody.

The indicated dose and times of the LPS administration were used to stimulate neutrophils with or without AMP. The neutrophils were preincubated with the A1 receptor antagonist, ecto-5′-nucleotidase inhibitor and p38 MAPK inhibitor for 30 min before the above treatments.

### ROS Measurement

Human and mouse neutrophil ROS measurements were performed using fluorescent detection of ROS activity by flow cytometry as previously described (15). Neutrophils were resuspended in RPMI 1,640. The cell suspension was pretreated with or without an inhibitor for 30 min before LPS and AMP stimulation. DHR 123 was added to the cells at a final concentration of 1 μM for 15 min. The reaction was stopped on ice for 5 min, and then the cells were washed twice with ice-cold PBS. ROS activity in each group was immediately measured by flow cytometry and analyzed by FlowJo software.

### Adhesion Measurement

A 96-well plate was precoated with 20 μl FBS. Then, neutrophils (10^5^ in 100 μl) were seeded into the wells with the indicated treatments and cultured for 1 h at 37°C with 5% CO_2_. Neutrophils were fixed with 2% paraformaldehyde for 15 min. The unbound neutrophils were washed away, and adherent neutrophils were stained with DAPI. The mean fluorescence intensity was detected.

### Phagocytosis Assay

The phagocytic activity of neutrophils was assayed by using a pHrodo *E. coli* Bioparticles Phagocytosis Kit (Thermo Fisher, Waltham, Massachusetts, US). Neutrophils from each group were collected and washed twice with ice-cold PBS. Neutrophils were treated as indicated and then mixed with pHrodo *E. coli* and incubated for 1 h at 37°C. The engulfed bacteria display fluorescence when in the low pH environment of the acidified phagocytic compartment. Phagocytosis was determined by flow cytometry within 1 h.

### Apoptosis

For the apoptosis assay, an apoptosis detection kit (Vazyme Biotech, Jiangsu, China) was used according to the manufacturer's instructions. Briefly, mouse bone marrow neutrophils were treated as indicated for 4 h, collected, washed twice, and resuspended in 1 × binding buffer at a concentration of 1 × 10^6^ cells/ml. Then, 5 μl Annexin V and 5 μl PI were added to the cell suspension, and the samples were incubated for 15 min in the dark. Apoptosis was determined by flow cytometry, and apoptotic cells were Annexin V-positive and PI-negative/positive.

### Degranulation

Degranulation analysis of neutrophils was performed by flow cytometry after the neutrophils had been treated as indicated. Degranulation of secretory vesicles, specific granules, and azurophil granules was determined by measuring the increase in plasma membrane expression of surrogate markers using the following mAbs: PE-conjugated anti-human CD63 (clone: H5C6), FITC-conjugated anti-human CD66b (clone: G10F5), FITC-conjugated anti-human CD35 (clone: E11), and PE-conjugated anti-mouse CD63 (clone: NVG-2). The cells were analyzed by flow cytometry and quantified by using FlowJo software as previously described.

### Western Blotting

Mouse neutrophils (treated as indicated) were collected to examine protein expression. Briefly, total cell lysates were obtained and mixed with 3 × SDS buffer, boiled, and loaded on 10% SDS-PAGE gels. Equal amounts of protein were separated by SDS-PAGE and transferred to nitrocellulose filters. Non-specific binding was blocked with 3% BSA in TBS/Triton, followed by incubation with primary antibodies at 4°C overnight. Then, the membranes were incubated with the appropriate secondary antibody for 1 h before enhanced chemiluminescence detection. The bands were visualized using an ECL reagent.

### Proteome Arrays

The Proteome Profiler™ Array was performed according to the manufacturer's instructions (R&D System, Minneapolis, MN, USA) and was used to determine the relative phosphorylation levels of serine/threonine and receptor tyrosine kinases. Neutrophils were divided into three groups: control (vehicle-treated cells; PBS, used for dissolution of LPS), LPS, and LPS + AMP. After stimulation for 45 min, the cells were collected for the proteome arrays. Briefly, total protein was extracted using lysis buffer. 300-μg cell lysates were diluted, mixed with a cocktail of biotinylated detection antibodies, and incubated overnight with the proteome array membrane at 4°C. Then, the membrane was washed with 1 × wash buffer for three times and incubated with streptavidin-HRP for 30 min at room temperature. The membrane was washed three times, and 1 mL of the Chemi Reagent Mix was pipetted evenly onto the membrane. Finally, the membrane was exposed to X-ray film for 3 min. The pixel density was determined with ImageJ software.

### AMP and ADO Detection

The quantitative determination of AMP and ADO was performed by using an AMP or ADO Assay Kit according to the manufacturer's instructions (BioVision). Blood samples from septic patients and healthy volunteers were collected. Serum samples and the cell culture supernatant were used for AMP or ADO detection.

In this assay, AMP is converted to pyruvate in the presence of pyrophosphate and phosphoenolpyruvate. This is followed by a set of enzymatic reactions to generate a colored product with a strong absorbance at 570 nm. The absorbance is proportional to the amount of AMP present in samples. Briefly, samples were mixed with Reaction Mix 50 μL (AMP enzyme, AMP developer, AMP substrate mix, and AMP probe and AMP assay buffer), and the well volume was adjusted to 100 μL with the AMP assay buffer. Then, incubation was done at 37°C for 60 min and OD was measured at 570 nm. A standard curve is plotted relating the intensity of the color (OD) to the concentration of standards. The AMP concentration in each sample is interpolated from this standard curve.

In terms of ADO detection, samples were mixed with Reaction Mix 50 μL (ADO detector, ADO convertor, ADO developer, and ADO probe and ADO assay buffer) and the well volume was adjusted to 100 μL with ADO assay buffer. Then, incubation was done at 37°C for 15 min and fluorescence (Ex/Em = 535/587 nm) measured in a plate reader.

### Statistical Analysis

All statistical analyses were performed with GraphPad Prism Software (version 4). Continuous data with a normal distribution are expressed as the mean ± SD. Those with a non-normal distribution are expressed as the median. Continuous data were analyzed using one-way ANOVA for multiple groups and Student's *t*-test for 2 groups. Correlations were assessed using the Spearman rank test. Logistic regression and analysis were performed to evaluate the relationship between the relevant variables. Statistical significance was set at *p* ≤ 0.05.

## Results

### AMP Inhibits Neutrophil Infiltration in LPS-Induced Endotoxemic Mice and Alleviates Tissue Damage

**Figure 1:**
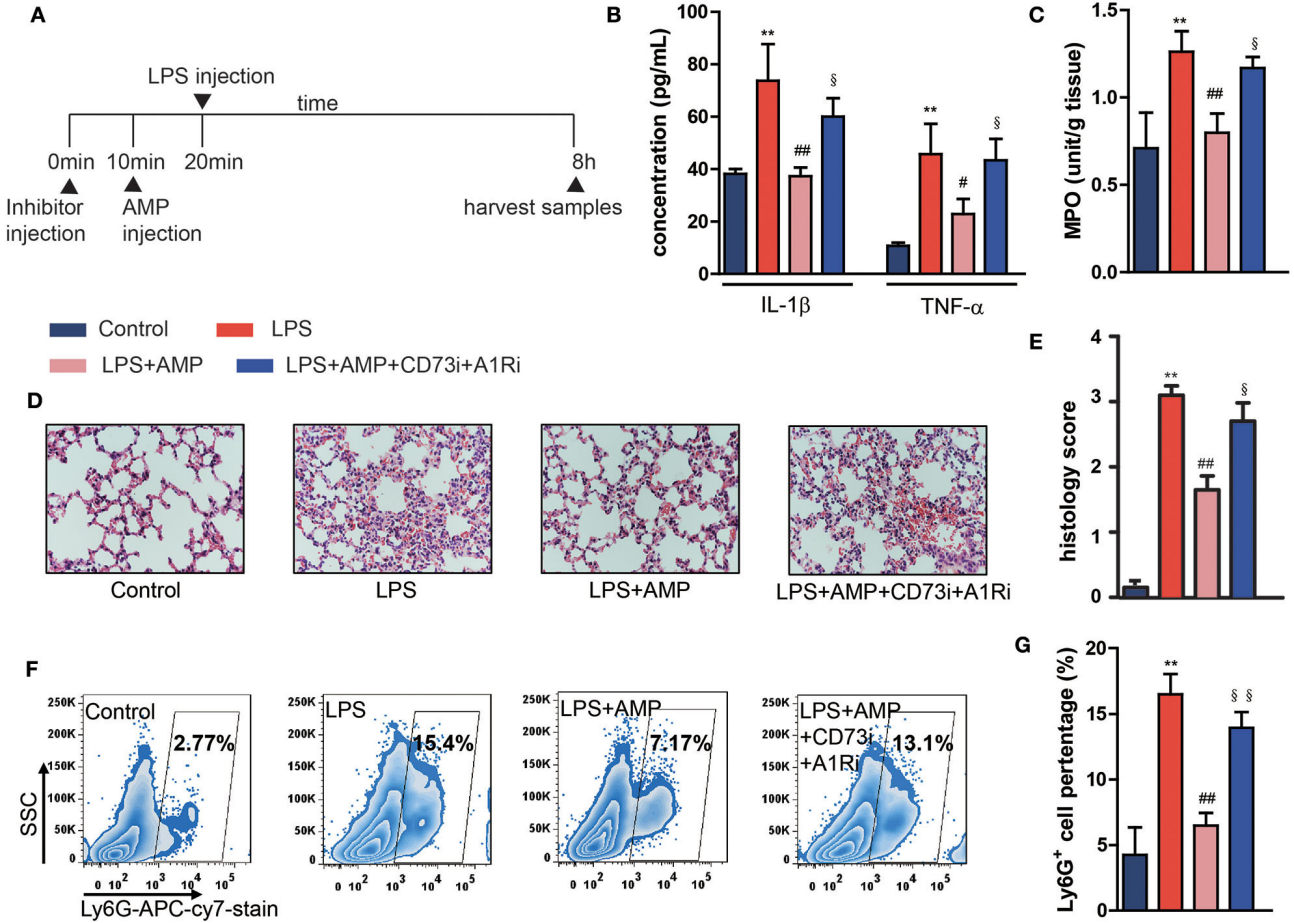
AMP inhibits neutrophil infiltration in LPS-induced endotoxemic mice and alleviates tissue damage. C57BL/6 mice were pretreated with or without the A1R antagonist and ecto-5′-nucleotidase inhibitor, followed by LPS or AMP injections or PBS. The mice were euthanized 8 h after LPS treatment, and serum and lung tissues were collected for assessment. **(A)** Sketch map of *in vivo* treatments. **(B)** Levels of serum IL-1β and TNF-α were measured with ELISA kit. **(C)** Lung MPO activity. **(D)** Pathological sections of the lung 8 h after LPS injection. **(E)** The histopathologic scores are presented for the lung tissues. **(F,G)** Neutrophil infiltration in the lung was detected by flow cytometry. The data are expressed as the mean ± SD, *n* = 6 for each group. ^*^P < 0.05, ^**^P < 0.01 compared to the control group, ^#^*P* < 0.05, ^*##*^*P* < 0.01 compared to the LPS group; ^§^*P* < 0.05, ^§§^*P* < 0.01 compared to the LPS + AMP group.

To investigate the effects of extracellular AMP on LPS-induced endotoxemia, C57BL/6 mice were intraperitoneally injected with LPS (10 mg/kg) and treated with AMP (50 mg/kg). Serum cytokines (TNF-α and IL-1β) were markedly increased after LPS injection (Figure 1B). Treatment with AMP significantly reduced the levels of TNF-α and IL-1β in LPS-challenged mice (Figure 1B). In addition, MPO activity was increased following stimulation with LPS, while treatment with AMP abolished the increased MPO activity (Figure 1C). H&E staining of lung sections showed that in LPS-induced endotoxemic mice, the lungs displayed pulmonary edema, expansion of pulmonary capillaries, red blood cell extravasation, and apparent neutrophil infiltration. AMP protected the lung from injury and the increase in neutrophil infiltration (Figure 1D). Moreover, histological lung injury scores are presented (Figure 1E). Consistent with H&E staining, flow cytometry showed that LPS administration increased the percentage of neutrophils in the lung, while AMP treatment decreased this percentage (Figures 1F,G). These results indicate that AMP suppresses inflammation in an LPS-induced mouse model.

### AMP Inhibits Neutrophil Activation in an A1R-Dependent Manner

**Figure 2:**
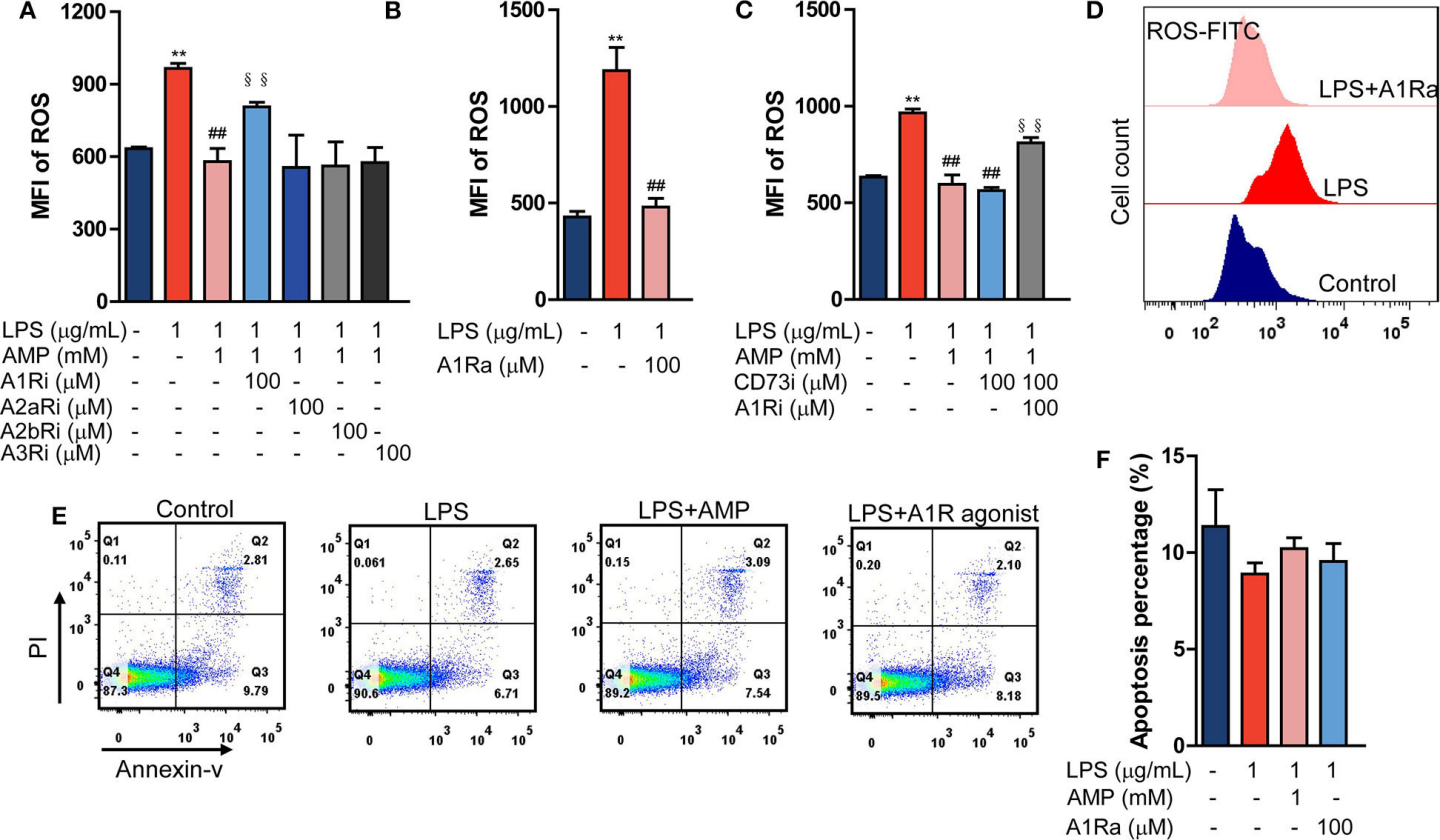
AMP inhibits neutrophil activation in an A1R-dependent manner. **(A)** LPS stimulation neutrophil treated with AMP and different P1 receptor antagonists, ROS levels were detected using flow cytometry. **(B,D)** LPS stimulation neutrophil treated with A1R agonist; ROS levels were measured. **(C)** LPS stimulation neutrophil treated with A1R antagonist and ecto-5′-nucleotidase inhibitor; ROS production was shown. **(D)** Representative images of flow cytometry showing ROS levels within 1 h. **(E)** The apoptotic rate was measured in the LPS, LPS+AMP, and LPS+A1R agonist groups. **(F)** Statistical analyses of neutrophil apoptosis. The data are expressed as the mean ± SD, *n* = 6 for each group. ^*^P < 0.05, ^**^P < 0.01 compared to the control group; ^#^*P* < 0.05, ^*##*^*P* < 0.01 compared to the LPS group; ^§^*P* < 0.05, ^§§^*P* < 0.01 compared to the LPS + AMP group.

Data showed that LPS stimulation enhanced the neutrophil ROS level. While being treated with dose-dependent AMP, both 0.1 mM and 1 mM were sufficient to inhibit LPS-induced neutrophil ROS generation, and 1 mM had a better effect (Supplementary Figure 1A). Therefore, for *in vitro* mechanistic studies, 1 mM of AMP was used. P2 receptor-associated purinergic signaling prevalently facilitates neutrophil activation, whereas P1 receptor-associated purinergic signaling mostly restricts neutrophil activation (11); therefore, we hypothesized that P1 receptors (including A1R, A2aR, A2bR, and A3R) contribute to the inhibition of endotoxemic inflammatory responses and neutrophil activation. It has been shown that P2 inhibitors cannot abolish the effect of AMP (Supplementary Figure 2). Then, by using specific antagonists of A1R, A2aR, A2bR, and A3R, we found that only the A1R antagonist counteracted the suppression of AMP on LPS-induced neutrophil ROS production (Figure 2A, Supplementary Figure 1B). In addition, an A1R agonist inhibited ROS production in LPS-stimulated neutrophils (Figures 2B,D). Of note, because extracellular AMP can be hydrolyzed to ADO by CD73 and ADO suppresses inflammatory responses, we applied a CD73 inhibitor to eliminate the effects of AMP hydrolysis. Data showed that the CD73 inhibitor failed to reverse the effects of AMP (Figure 2C, Supplementary Figures 1C–D). In addition, to further confirm that the CD73 inhibitor used was functional, extracellular AMP and ADO levels were measured. Supplementary Figure 1E showed that the CD73 inhibitor significantly suppressed the conversation of AMP to ADO, which all indicated that the role of AMP in inhibiting LPS-induced neutrophil ROS production was not dependent on the hydrolysate ADO. To exclude the possibility that the suppressive effect of AMP and the A1R agonist were the result of impaired cell viability, Annexin V-PI staining was performed. As shown in Figures 2E,F, treatment with AMP and the A1R agonist did not affect LPS-stimulated neutrophil viability compared with that of the LPS group. Furthermore, we found that in LPS-induced endotoxemic mice, treatment with the CD73 inhibitor + A1R antagonist reversed the effects of AMP (Figures 1B–G). Together, these findings suggest that AMP alone suppresses neutrophil activation and septic inflammatory responses by activating A1R.

### Extracellular AMP Regulates LPS-Stimulated Mouse Neutrophil Functions

**Figure 3:**
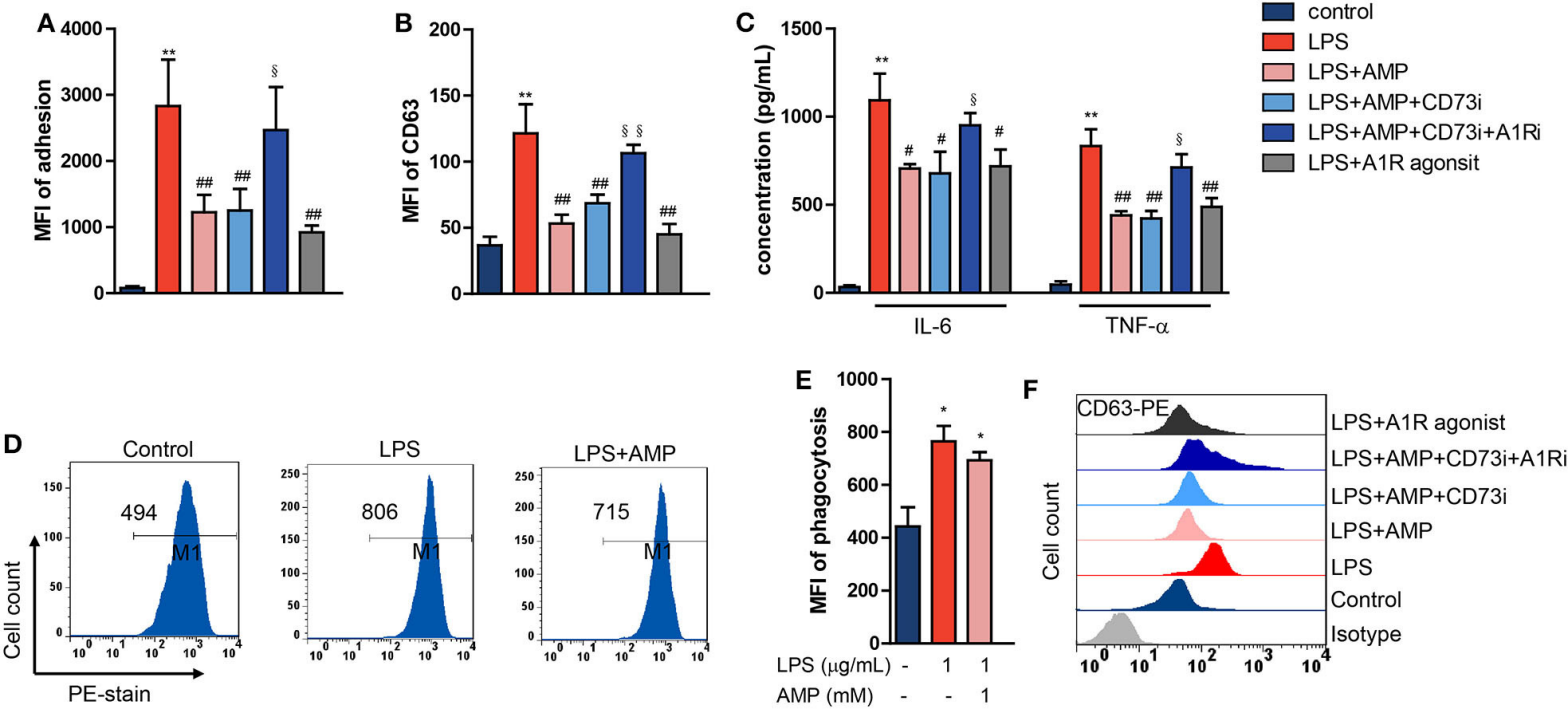
Extracellular AMP regulates LPS-stimulated mouse neutrophil functions. LPS stimulation neutrophil treated with AMP, A1R agonist, A1R antagonist, and ecto-5′-nucleotidase inhibitor. Neutrophil adhesion **(A)**, neutrophil degranulation (CD63) **(B,F)**, neutrophil TNF-α and IL-6 production **(C)**, and neutrophil phagocytic activity **(D)** were detected. **(E)** Statistical analyses of neutrophil phagocytosis. **(F)** Representative images of flow cytometry showing CD63 levels. The data are expressed as the mean ± SD, *n* = 6 for each group. ^*^P < 0.05, ^**^P < 0.01 compared to the control group; ^#^*P* < 0.05, ^*##*^*P* < 0.01 compared to the LPS group; ^§^*P* < 0.05, ^§§^*P* < 0.01 compared to the LPS + AMP group.

To further confirm our findings that AMP alone inhibits neutrophil activation in an A1R-dependent manner, neutrophil functions, including degranulation, adhesion, cytokine production, and phagocytic activity, were evaluated. We found that LPS stimulation promoted neutrophil adhesion and degranulation, and AMP reduced the increase in adhesion and degranulation, an effect that was abolished by the CD73 and A1R antagonists (Figures 3A,B,F). Similarly decreased adhesion and degranulation were observed in response to the A1R agonist (Figures 3A,B,F). Concentrations of cytokines were detected in supernatants of neutrophils that were stimulated as indicated. TNF-α and IL-6 production in LPS-challenged neutrophils was inhibited by AMP, and the effect was A1R-dependent (Figure 3C). Then, we tested the effect of AMP on the phagocytic activity of LPS-stimulated neutrophils. The data showed that LPS stimulation enhanced neutrophil phagocytic activity (Figures 3D,E), which was consistent with previous reports. AMP did not affect neutrophil phagocytic activity (Figures 3D,E). Taken together, these results suggest that LPS-induced neutrophil activation, as indicated by degranulation, adhesion, and cytokine secretion, was reversed by AMP via A1R.

### AMP Inhibits Neutrophil Activation by Interfering With the p38 MAPK Signaling Pathway

**Figure 4:**
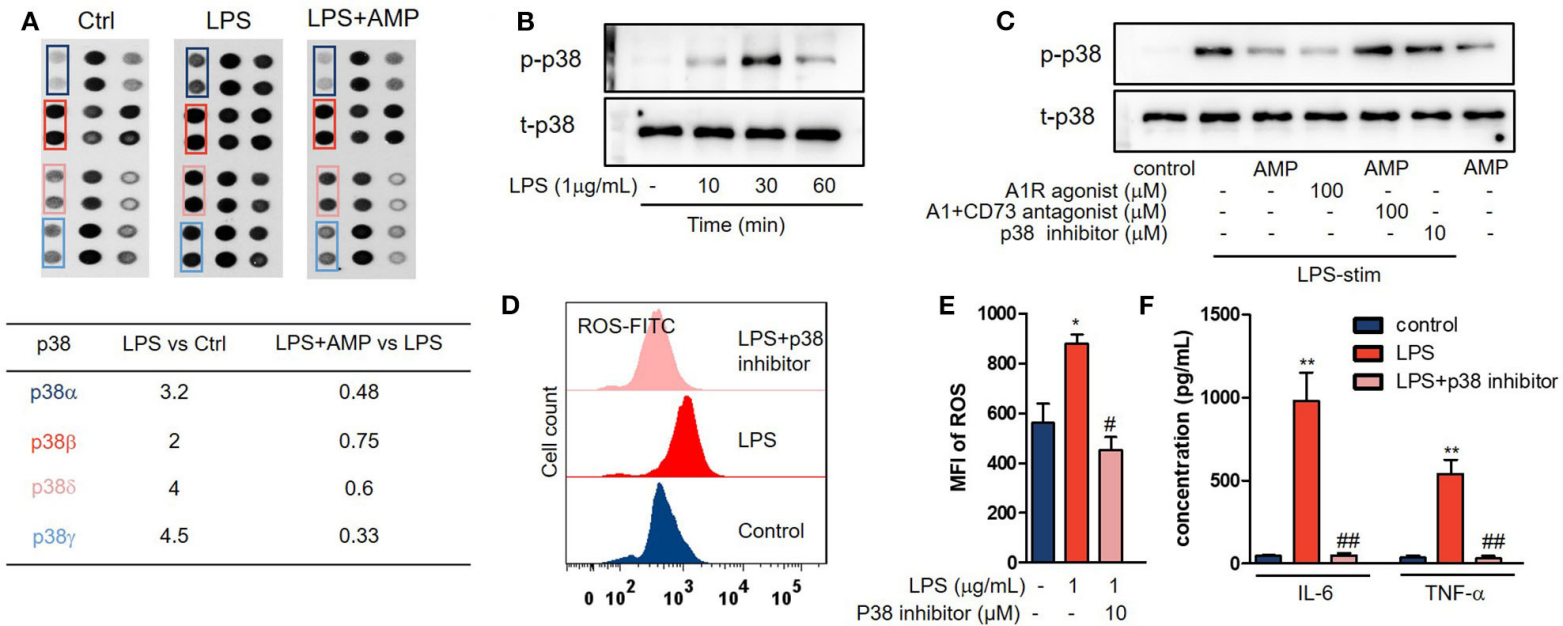
AMP inhibits neutrophil activation by interfering with the p38 MAPK signaling pathway. **(A)** The Proteome Profiler™ array of neutrophils with the indicated treatments (control, LPS, and LPS + AMP) was shown. **(B)** Neutrophils treated with LPS in the indicated time; p38 MAPK phosphorylation was measured with western blot. **(C)** LPS stimulation neutrophil treated with AMP, A1R agonist, A1R antagonist, and ecto-5′-nucleotidase inhibitor and p38 inhibitor; p38 MAPK phosphorylation was measured. Neutrophils stimulated with the indicated treatments (control, LPS, and LPS+p38 inhibitor), ROS levels **(D,E)**, and TNF-α and IL-6 production **(F)** were detected. **(E)** Statistical analyses of neutrophil ROS levels. The data are expressed as the mean ± SD, *n* = 6 for each group. ^*^P < 0.05, ^**^P < 0.01 compared to the control group; ^#^*P* < 0.05, ^*##*^*P* < 0.01 compared to the LPS group; ^§^*P* < 0.05, ^§§^*P* < 0.01 compared to the LPS + AMP group.

To explore the mechanisms by which AMP inhibits neutrophil activation, we performed a phospho-MAPK proteome array. As shown in Figure 4A, LPS treatment increased the phosphorylation level of p38 MAPK. However, treatment with AMP markedly decreased p38 MAPK phosphorylation. Western blotting further confirmed significant p38 MAPK phosphorylation after LPS stimulation at 10, 30, and 60 min (Figure 4B). AMP or A1R agonist intervention inhibited p38 MAPK phosphorylation. In the presence of the A1R antagonist and CD73i, AMP-induced suppression of p38 MAPK phosphorylation was abolished (Figure 4C). SB203580 is a selective inhibitor of p38 MAPK that inhibits p38 catalytic activity by binding to the ATP-binding pocket but does not inhibit the phosphorylation of p38 by upstream kinases. As a negative control, we found that SB203580 failed to affect p38 phosphorylation as previously described (Figure 4C) (16). Next, ROS generation and cytokine production were determined to further confirm that AMP-A1R-mediated suppression of neutrophil activation was p38 MAPK-dependent. As shown in Figures 4D–F, LPS stimulation increased the production of ROS and cytokines (IL-6 and TNF-α). However, SB203580 reversed this effect. Representative images of ROS are shown in Figure 4D.

### The Role of AMP in LPS-Stimulated Human Neutrophils

**Figure 5:**
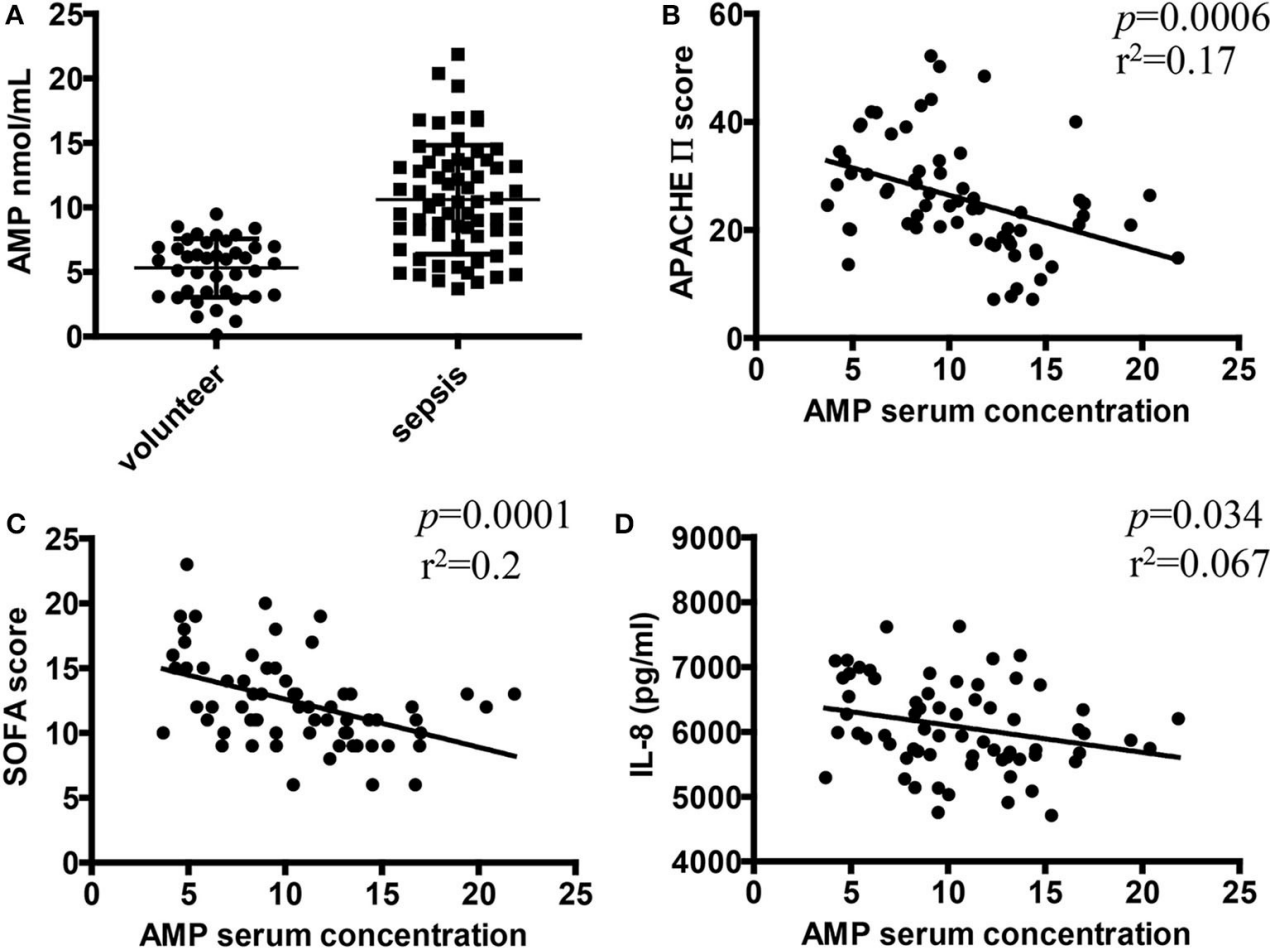
The role of AMP in LPS-stimulated human neutrophils. After pretreatment with or without the A1R antagonist and ecto-5′-nucleotidase inhibitor, human neutrophils from healthy volunteers or septic patients were incubated with or without LPS or AMP. The ROS level **(A)**, neutrophil phagocytic activity **(B)**, and CD66b, CD63, and CD35 expression **(C–E)** were detected by using flow cytometry. The data are expressed as the mean ± SD, *n* = 5 for each group. ^&^*P* < 0.05, ^&&^*P* < 0.01 compared to the volunteer group; ^*^P < 0.05, ^**^P < 0.01 compared to the control group; ^#^*P* < 0.05, ^*##*^*P* < 0.01 compared to the LPS group; ^§^*P* < 0.05, ^§§^*P* < 0.01 compared to the LPS + AMP group.

The above results indicated that extracellular AMP regulates mouse neutrophil function via occupation of A1R. To determine whether AMP exerts a similar effect on human neutrophils, primary neutrophils were obtained from healthy volunteers and septic patients and treated with LPS in the presence or absence of AMP. The isolated neutrophils were exposed to LPS, and then ROS generation and phagocytosis were examined. The sepsis group showed increased ROS generation and phagocytosis in response to LPS stimulation compared with the volunteer group (Figures 5A,B). We further examined whether neutrophils isolated from patients with sepsis were primed for increased granule release in response to LPS stimulation. To assess degranulation, the surface expressions of azurophilic granules (CD63), specific granules (CD66b), and secretory vesicles (CD35) were examined. The basal expression levels of CD63 and CD35 were significantly increased in the sepsis group compared with those in the volunteer group (Figures 5C–E). After LPS stimulation, the sepsis group had higher levels of CD63 and CD35 surface expression than those of the volunteer group (Figures 5C–E). Further, when treated with AMP, the enhanced ROS and degranulation were suppressed (Figures 5A,C–E). However, these effects were reversed in the presence of the A1R antagonist and CD73i (Figures 5A,C–E). These results indicate that AMP inhibits the activation of LPS-stimulated neutrophils in an A1R-dependent manner.

### Correlations Between Serum AMP Level and Inflammatory Disease Severity in Patients With Sepsis

**Figure 6:**
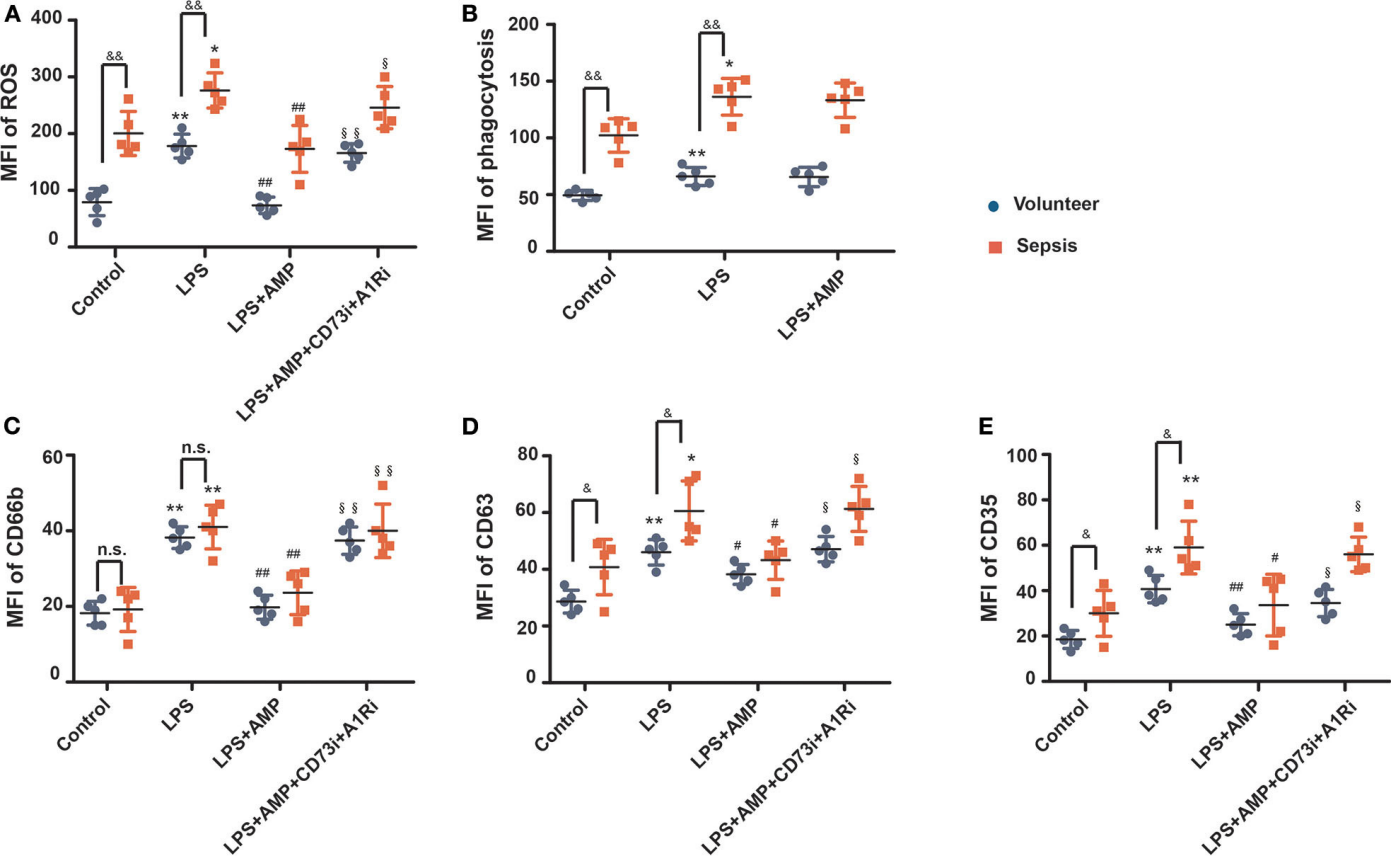
Correlations between serum AMP level and inflammatory disease severity in patients with sepsis. **(A)** The serum level of AMP in patients with sepsis and healthy controls. **(B,C)** Relationship between serum AMP level and disease severity (correlation of AMP level with scores of Acute Physiology and Chronic Health Evaluation (APACHE) and Sequential Organ Failure Assessment (SOFA) by linear regression analysis). **(D)** Relationship between serum AMP and inflammation cytokine IL-8. ^*^P < 0.05, ^**^P < 0.01 compared to the control group; ^#^*P* < 0.05, ^*##*^*P* < 0.01 compared to the LPS group; ^§^*P* < 0.05, ^§§^*P* < 0.01 compared to the LPS+AMP group. n.s., stands for non-significantly.

Our studies showed that AMP inhibited inflammatory responses and neutrophil activation in a murine endotoxemia model. However, the potential roles of AMP in clinical septic patients remain unknown. Therefore, a total of 67 patients with sepsis (Table 1) and 40 healthy volunteers were recruited in this study to evaluate the relationship between AMP and sepsis. As shown in Figure 6A, the mean AMP serum level was significantly higher in patients with sepsis than in healthy controls. Then, we further investigated the correlation between AMP concentration and inflammatory disease severity, including inflammation cytokine IL-8, and APACH score and SOFA score, both of which are associated with inflammatory disease severity. As shown in Figures 6B–D, the serum level of AMP was negatively correlated with the APACH score, SOFA score, and IL-8 level, which indicated that AMP acts as a protective molecule in sepsis.

**Table 1:** Patients characteristics.

		**Septic**
Patient		67
Age		
	Median	67
	IQR	56–75
Gender		
	Male	31
	Female	36
APACHE II		
	Median	24
	IQR	18–32
SOFA (sequential organ failure assessment)		
	Median	13
	IQR	11–16
ALC (absolute lymphocyte count) (cells × 10^3^/microliter)		
	Median	0.5
	IQR	0.4–0.8
ANC (absolute neutrophil count) (cells × 10^9^/microliter)		
	Median	12.8
	IQR	6.55–16.98
INR (international normalized ratio) (seconds)		
	Median	1.3
	IQR	1.16–1.49
Serum creatinine (mg/dl)		
	Median	114
	IQR	78.35–194.98
Length of ICU stay		
	Median	5
	IQR	3–9
28-day mortality (%)		
	Survived	50
	Expired	17
Admission ICU diagnosis		
	Peritonitis	15
	Wound infection	4
	Line infection	30
	Community-aquired pneumonia	8
	Ventilator-associated pneumonia	3
	Trama	0
	Inta-cranial hemorrhage	0
	post-op(major surgery)	0
Co-morbidities		
	Diabetes	21
	Heart disease	15
	Morbid obesity	2
	Neurologic	6
	Renal disease	15
	Respiratory	11
	Liver	17

## Discussion

Intracellular AMP modulates diverse biological processes, such as energy metabolism, cancer, cardiovascular disease, and inflammation, by activating AMPKs (17). However, the effect of extracellular AMP remains unclear. Sepsis is a life-threatening organ dysfunction resulting from a dysregulated host response to infection (18). Neutrophils are the first line of host defense and play a critical role in infection elimination (19, 20). However, overwhelming neutrophil activation can be harmful to the host by causing collateral tissue damage (21). In this study, LPS stimulation has been performed to mimic the septic inflammatory responses via TLR4 receptor activation, which could enable us to study the specific mechanism in a relatively concise condition. Then, we performed a series of experiments to determine the suppressive effect of AMP on endotoxemic-induced inflammation and neutrophil activation. Our results showed that AMP significantly alleviated tissue damage and suppressed the infiltration of neutrophils in lung tissues. Additionally, inflammatory cytokines (TNF-α and IL-1β) were also suppressed by AMP.

Exposure of murine bone marrow neutrophils to LPS led to dramatic ROS production. A large amount of ROS leads to tissue damage by oxidative stress and DNA damage. Our data revealed that AMP significantly decreased LPS-induced ROS production in murine bone marrow neutrophils. AMP is readily hydrolyzed to ADO by neutrophil-expressed CD73 (22). Because of the identical abilities of ADO and AMP to activate A1Rs (23), we used a CD73 inhibitor to exclude the effect of ADO. Our data showed that the CD73 inhibitor failed to alter ROS production in AMP-treated LPS-stimulated neutrophils, which indicated that AMP alone inhibits LPS-induced ROS production. Furthermore, AMP-mediated suppression was reversed by the A1R antagonist, and this was also confirmed by using the A1R antagonist and CD73 inhibitor together, indicating the involvement of A1R in mediating the AMP effect. In addition, the data showed that the inhibitory effect was also achieved by the A1R agonist. Therefore, we hypothesized that AMP counteracts LPS-induced neutrophil activation via A1R. To address this issue, we further evaluated the effects of extracellular AMP on neutrophil adhesion, degranulation, phagocytic activity, and cytokine production after LPS stimulation. As one of the first inflammatory cells to migrate to inflammatory sites, neutrophils are activated to contain pathogens (24, 25). Overwhelming neutrophil activation causes severe tissue damage, multiple-organ failure, or death. LPS induced the activation of neutrophils, as shown by increased cytokine production and degranulation. However, when treated with AMP, the above increases in degranulation and cytokine production were decreased, suggesting a suppressive role of AMP on neutrophil activation. In addition, neutrophil adhesion, which is crucial to migration, was enhanced by LPS stimulation. During sepsis, increasing the adhesion of neutrophils leads to enhanced interactions between neutrophils and endothelial cells followed by vascular endothelial injury and organ dysfunction (26). Our results demonstrated that AMP treatment reduced adhesion and that this effect was A1R-dependent. AMP did not alter the phagocytic activity of LPS-stimulated neutrophils, which suggested that AMP will not affect neutrophil phagocytosis of pathogens to protect against endotoxemia. Noteworthily, A1R is expressed on various immune cells, including monocytes, and macrophages, indicating that AMP may also regulate function of other immune cells in endotoxemia (27). In the present study, we focused on the specific biological functions of extracellular AMP on neutrophils. Further studies are still needed to clarify the effects of AMP on other immune cells.

LPS-triggered activation of p38 MAPKs is a critical signal transduction pathway that modulates various neutrophil functions (28, 29). In the present study, we demonstrated that AMP inhibited the phosphorylation of p38 MAPK in LPS-activated neutrophils. Similar results were observed when the cells were treated with the A1R agonist. Adding an A1R antagonist before LPS and AMP treatment reversed the AMP-mediated decreases in p38 MAPK phosphorylation. The intracellular signaling pathways downstream of A1R mediating p38 MAPK phosphorylation have not been elucidated. A1R is coupled to G protein. Subsequent to activating A1R, signaling cascades are initiated, and the most-characterized mechanism is the effect on adenylate cyclase. The activation of MAPK by G protein-coupled receptors can occur by several mechanisms. This activation may be dependent on or independent of PKA, PKC, Src tyrosine kinase, or Ras activation and involves the cross-activation of receptor tyrosine kinases (30). The previous study showed that in the smooth muscle cell line, activation of A1R is associated with the phosphorylation of p38 MAPK. It seems likely that regulating p38 MAPK by the adenosine A 1 receptors is mediated by G i/o proteins coupling to classical intracellular signaling pathways such as modulation of cAMP production or the phospholipase C pathway (31). Whether the signal transduction mediated the neutrophil function is unclear, which needs more future studies on this issue. Then, we used a p38 MAPK inhibitor to further verify the mechanism. The p38 MAPK inhibitor abolished ROS and inflammatory cytokine production in LPS-stimulated neutrophils. Consistent with the effects on inflammatory cytokines and ROS, we suggest that AMP regulates LPS-stimulated neutrophil function via inhibition of the p38 MAPK signaling pathway.

We further investigated the relevance of our findings in human neutrophils. We demonstrated here that AMP regulates human neutrophil functions (ROS production, degranulation, and phagocytic activity) in an A1R-dependent manner. Taken together, these results uncovered a role for AMP which may be of great importance in the regulation of neutrophil function during endotoxemia and may provide a novel strategy to address the uncontrolled neutrophil functions during endotoxemia. The clinical data further support the hypothesis that extracellular AMP is inhibitory during sepsis because serum AMP levels in septic patients were negatively correlated with inflammation marker (IL-8) and organ dysfunction scores (APACH and SOFA scores), which are all associated with sepsis severity.

We conclude that extracellular AMP contributes to the anti-inflammatory activity of LPS-induced septic mice by suppressing neutrophil activation. Furthermore, our study demonstrated an anti-inflammatory role for AMP by activating A1R and repressing p38 MAPK signaling in neutrophils after LPS stimulation. The serum level of AMP is associated with sepsis severity, which was observed in ICU patients. Further studies, however, are needed to elucidate its clinical translatability.

## Data Availability Statement

The raw data supporting the conclusions of this article will be made available by the authors, without undue reservation, to any qualified researcher.

## Ethics Statement

The studies involving human participants were reviewed and approved by Medical Ethical Committee of Jiangsu University. The patients/participants provided their written informed consent to participate in this study. The animal study was reviewed and approved by Medical Ethical Committee of Jiangsu University.

## Author Contributions

YH, DL, and DZ conducted experiments and drafted the manuscript. XW carried out the flow cytometry and helped to revise the manuscript. WQ and QW conceived of the study, helped to draft the manuscript and finalized the manuscript. All authors contributed to the article and approved the submitted version.

## Conflict of Interest

The authors declare that the research was conducted in the absence of any commercial or financial relationships that could be construed as a potential conflict of interest.

## Supplementary Material

Supplementary Figure 1 **(A)** The ROS level in dose response for AMP treatment was detected and analyzed. **(B,C)** LPS stimulation mouse neutrophil treated with AMP and different concentration of P1 receptor antagonists or CD73 inhibitor, ROS levels were detected using flow cytometry. **(D)** LPS stimulation human neutrophil treated with AMP and different concentration of CD73 inhibitor, ROS levels were detected. **(E)** Neutrophils with indicated treatments (AMP, AMP+CD73i) were incubated in 48-well plates with 100 μM AMP in 200 μl of incubation medium. An aliquot of the supernatant was withdrawn at 1 h and the presence of AMP and ADO were determined. The data are expressed as the mean ± SD, *n* = 6 for each group. ^*^*P* < 0.05, ^**^*P* < 0.01 compared to the control group or the AMP group; ^#^*P* < 0.05, ^*##*^*P* < 0.01 compared to the LPS group; ^§^*P* < 0.05, ^§§^*P* < 0.01 compared to the LPS+AMP group.

Supplementary Figure 2 LPS stimulation neutrophil treated with AMP and different concentration of indicated inhibitors, ROS levels were detected using flow cytometry. **(A)** CD39 inhibitor: POM1 **(B)** ADA inhibitor: EHNA **(C)** non-selective P2X inhibitor: PPADS **(D)** non-selective P2Y inhibitor: RB2. The data are expressed as the mean ± SD, *n* = 6 for each group. ^*^*P* < 0.05, ^**^*P* < 0.01 compared to the control group; ^#^*P* < 0.05, ^*##*^*P* < 0.01 compared to the LPS group.
