# Supplementary figures and images for: Extracellular AMP Suppresses Endotoxemia-Induced Inflammation by Alleviating Neutrophil Activation
Source: Front Immunol. 2020 Jul 7;11:1220. doi: 10.3389/fimmu.2020.01220 (PMC7358592; doi:10.3389/fimmu.2020.01220)

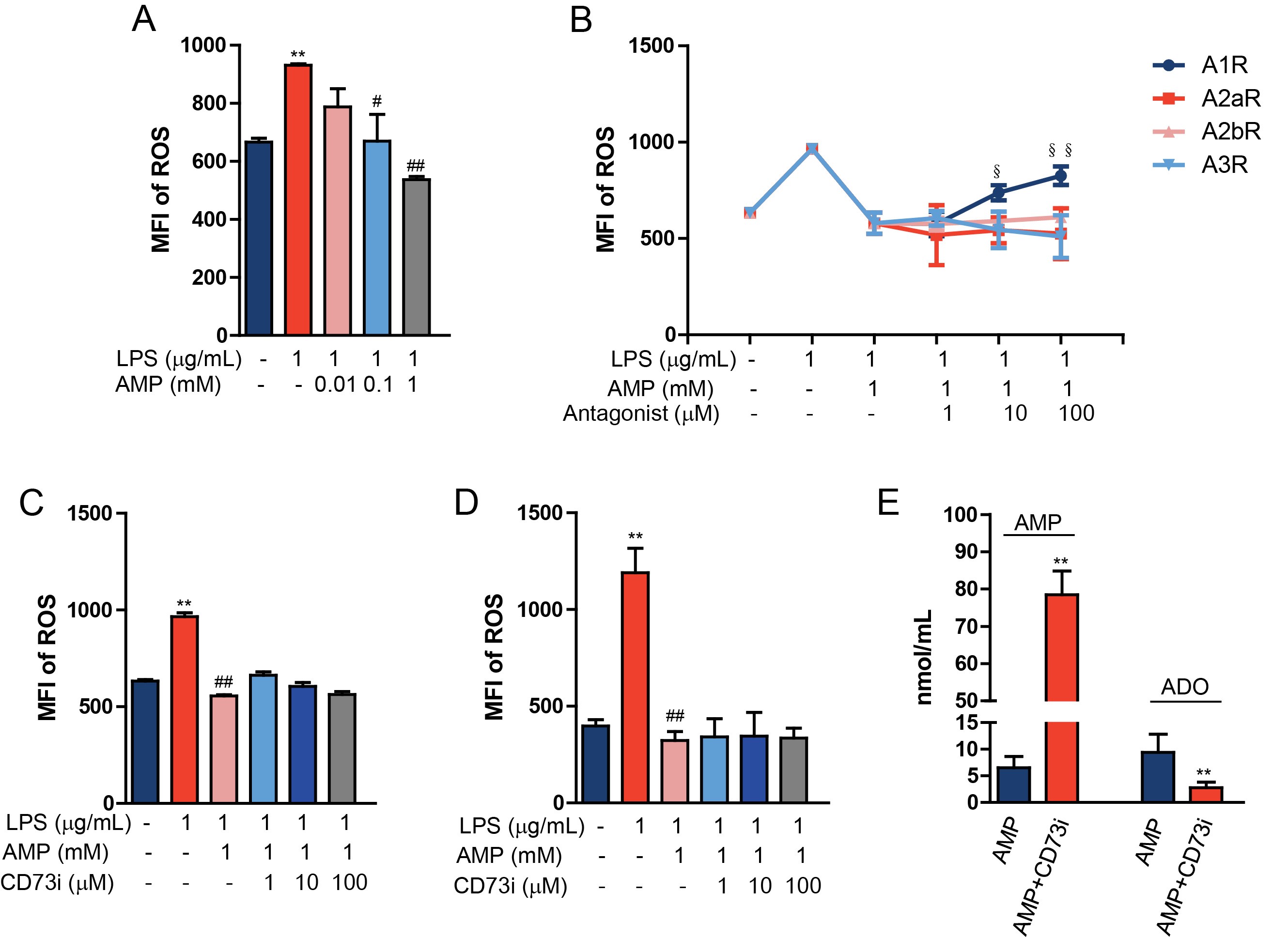

Supplement: Supplementary Figure 1 — (A) The ROS level in dose response for AMP treatment was detected and analyzed. (B,C) LPS stimulation mouse neutrophil treated with AMP and different concentration of P1 receptor antagonists or CD73 inhibitor, ROS levels were detected using flow cytometry. (D) LPS stimulation human neutrophil treated with AMP and different concentration of CD73 inhibitor, ROS levels were detected. (E) Neutrophils with indicated treatments (AMP, AMP+CD73i) were incubated in 48-well plates with 100 μM AMP in 200 μl of incubation medium. An aliquot of the supernatant was withdrawn at 1 h and the presence of AMP and ADO were determined. The data are expressed as the mean ± SD, n = 6 for each group. *P < 0.05, **P < 0.01 compared to the control group or the AMP group; #P < 0.05, ##P < 0.01 compared to the LPS group; §P < 0.05, §§P < 0.01 compared to the LPS+AMP group. [file Image_1.JPEG]

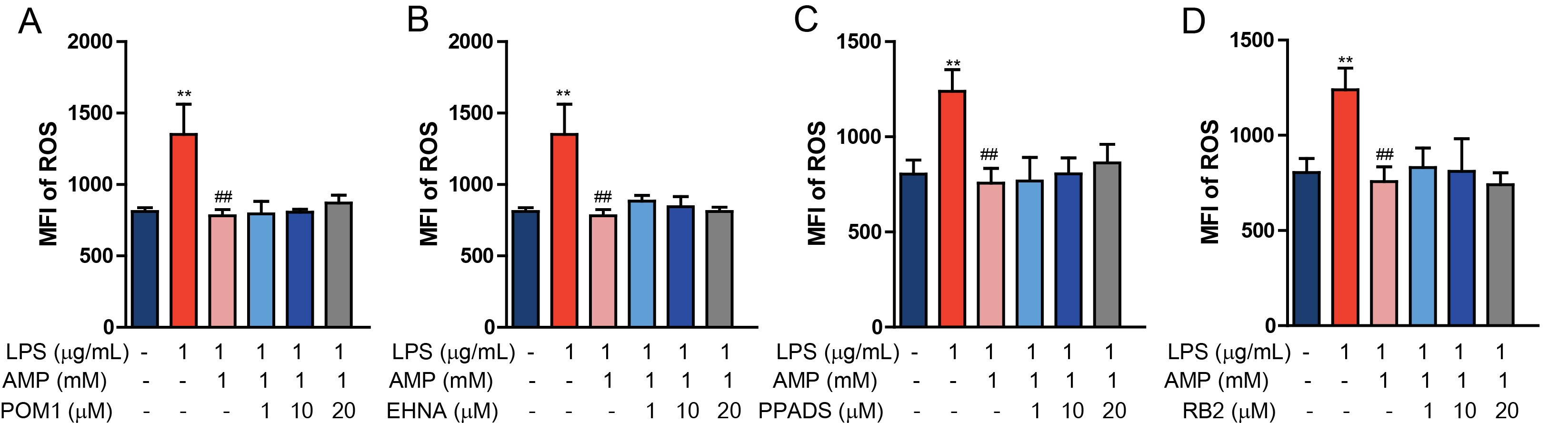

Supplement: Supplementary Figure 2 — LPS stimulation neutrophil treated with AMP and different concentration of indicated inhibitors, ROS levels were detected using flow cytometry. (A) CD39 inhibitor: POM1 (B) ADA inhibitor: EHNA (C) non-selective P2X inhibitor: PPADS (D) non-selective P2Y inhibitor: RB2. The data are expressed as the mean ± SD, n = 6 for each group. *P < 0.05, **P < 0.01 compared to the control group; #P < 0.05, ##P < 0.01 compared to the LPS group. [file Image_2.JPEG]
